# Case Report: Maintenance Nivolumab in Complete Responder After Multimodality Therapy in Metastatic Pancreatic Adenocarcinoma

**DOI:** 10.3389/fimmu.2022.870406

**Published:** 2022-04-28

**Authors:** Shih-Hung Yang, Jen-Chieh Lee, Bang-Bin Chen, Sung-Hsin Kuo, Chiun Hsu, Li-Yuan Bai

**Affiliations:** ^1^ Department of Oncology, National Taiwan University Hospital, Taipei, Taiwan; ^2^ Graduate Institute of Oncology, National Taiwan University College of Medicine, Taipei, Taiwan; ^3^ Department of Pathology, National Taiwan University Hospital, Taipei, Taiwan; ^4^ Department of Medical Imaging and Radiology, National Taiwan University Hospital, Taipei, Taiwan; ^5^ National Taiwan University Cancer Center, National Taiwan University College of Medicine, Taipei, Taiwan; ^6^ Division of Hematology and Oncology, Department of Internal Medicine, China Medical University Hospital, Taichung, Taiwan

**Keywords:** pancreatic ductal adenocarcinoma, maintenance therapy, chemotherapy, nivolumab, case report

## Abstract

Maintenance therapy is rarely considered in pancreatic ductal adenocarcinoma (PDAC). We describe the case of a 57-year-old man with metastatic PDAC treated with an initially full but subsequently de-escalated dose of combination chemotherapy due to intolerance to neurotoxicity. After a complete response to combined radiofrequency ablation for the liver metastasis and radiotherapy for the pancreatic tumor was achieved, chemotherapy was terminated and maintenance therapy was applied: nivolumab plus cytokine-induced killer cell therapy initially and then a de-escalated dosing interval of nivolumab monotherapy subsequently. No adverse events occurred during nivolumab therapy for more than 2 years, and the patient remains disease-free. To date, this is the first report of maintenance nivolumab after successful multimodality therapy in metastatic PDAC.

## Introduction

Pancreatic ductal adenocarcinoma (PDAC) remains the most challenging neoplasm. Multiagent chemotherapy regimens—such as gemcitabine plus nab-paclitaxel or the combination of 5-fluorouracil, folinic acid, irinotecan, and oxaliplatin (FOLFIRINOX)—remain the mainstay of treatment for patients with metastatic PDAC who have good performance status (PS) (1, 2). Although multiagent chemotherapy has notable toxicities (1, 2), mitigating these toxicities in the face of a deteriorating condition during the disease course may eventually jeopardize patients by precluding adequate chemotherapy doses in any effective regimen. Furthermore, how patients with metastatic PDAC who develop an extremely rare durable complete response (CR) to multiagent chemotherapy can be chronically managed remains unknown.

In the absence of defective mismatch repair (dMMR) or a high tumor mutation burden (TMB), immune checkpoint inhibitors (ICIs) have demonstrated modest efficacy in chemotherapy-treated PDAC (3). In first-line settings, the addition of nivolumab or pembrolizumab to gemcitabine plus nab-paclitaxel has resulted in manageable toxicities with a greater disease control rate and duration of response than those achieved with chemotherapy alone (1, 4, 5).

Here, we report a case of metastatic PDAC on long-term maintenance nivolumab after achieving CR with multimodal therapy without toxicities.

## Case Presentation

**Figure 1:**
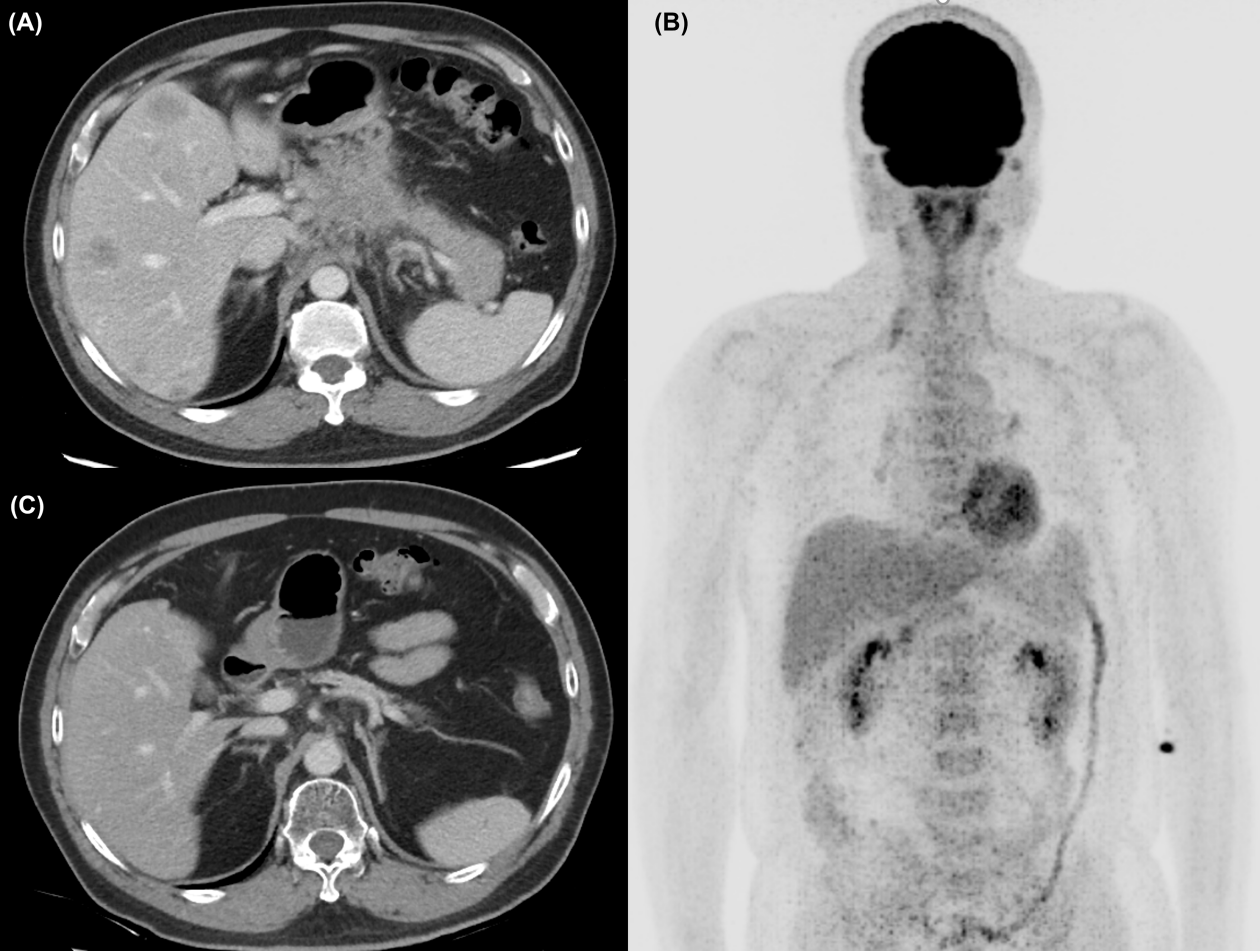
**(A)** The abdominal computed tomography (CT) scan revealed a 5-cm mass at the pancreatic body with liver metastases at diagnosis. **(B)** After chemotherapy and RFA of liver metastasis, no [18F]-FDG uptake was identified at PET scan. **(C)** At 32 months after initial diagnosis, the patient remains disease-free under maintenance nivolumab alone.

A 57-year-old man without any relevant medical history presented to our hospital in October 2018 with a 2-week history of epigastralgia. Mild epigastric tenderness was noted. The initial Eastern Cooperative Oncology Group PS was 0. Notably, his mother and sister had developed breast cancer at 80 and 42 years old, respectively. An abdominal computed tomography (CT) scan (**Figure 1A**) revealed a 5-cm mass at the pancreatic body with liver metastases and the staging of cT4N1M1. A liver biopsy revealed an adenocarcinoma with positive cytokeratin 7 staining through immunohistochemistry. The initial level of CA 19-9 was 62.7 U/ml.

Initially, the patient was enrolled in a clinical trial (NCT03162510) and treated with the SOLAR regimen consisting of oxaliplatin (75 mg/m^2^, day 1), nab-paclitaxel (150 mg/m^2^, day 1), S-1 (120 mg per day, days 1–7), and leucovorin in October 2018. Partial response was revealed by a CT scan after three cycles of chemotherapy. Dose de-escalation of chemotherapy was performed starting in the sixth cycle for grade 4 neutropenia. Eventually, he was withdrawn from the protocol-based chemotherapy after the 11th cycle due to intolerance to numbness. A CT scan in February 2019 demonstrated continuing shrinkage of the liver tumors and a nearly resolved pancreatic mass. He received radiofrequency ablation (RFA) for the residual liver tumor in April 2019, and reduced doses of the same chemotherapy agents were restarted concomitantly. After RFA, no [18F]-fluorodeoxyglucose uptake of liver tumors was identified through positron emission tomography (**Figure 1B**). During September and October 2019, radiotherapy to the pancreatic tumor (55 Gy) and regional lymphatic area (50 Gy) in 25 fractions was administered with reduced doses of the same chemotherapy agents.

**Figure 2:**
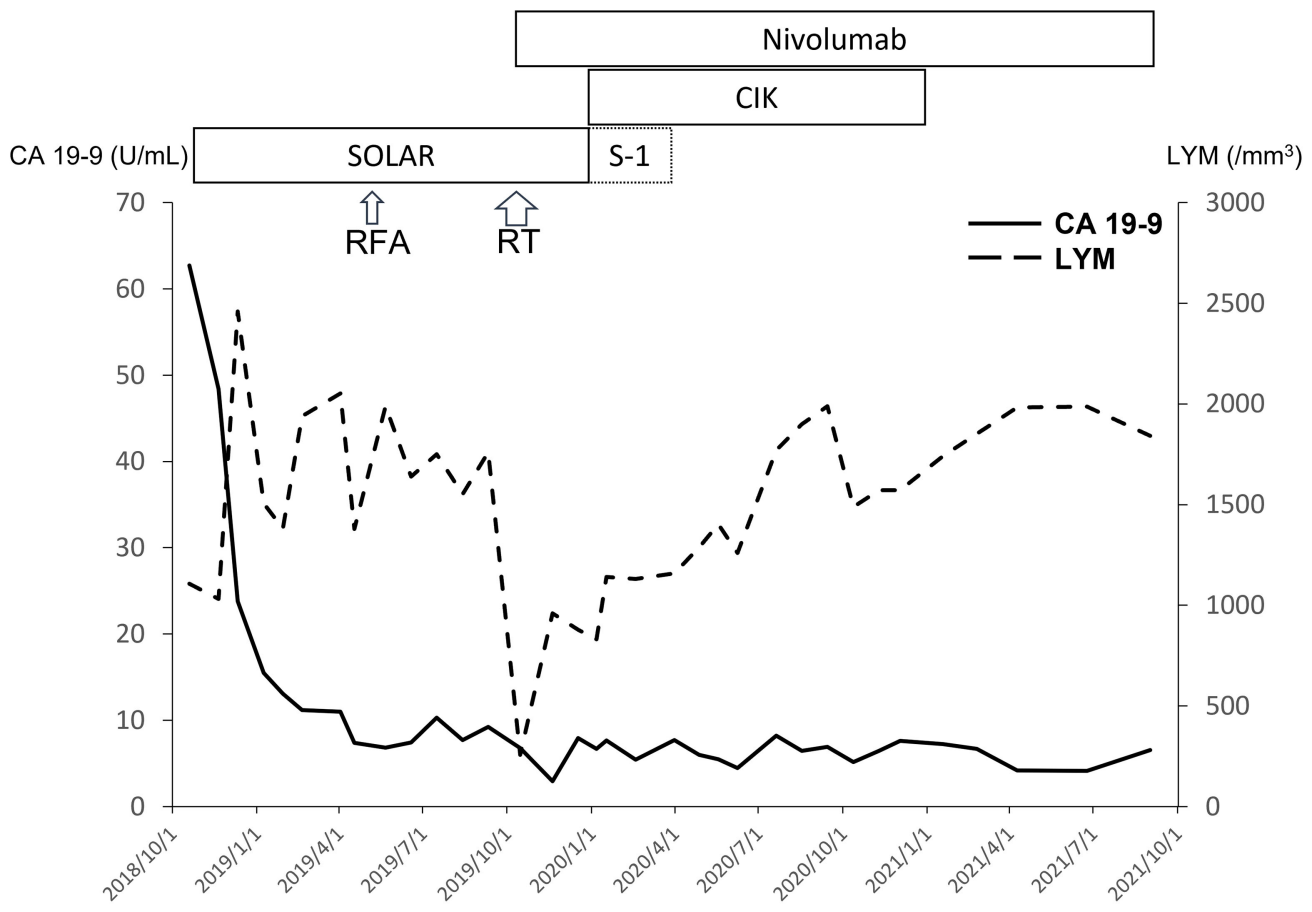
With extreme response to the SOLAR regimen, the level of CA 19-9 decreased rapidly. The lymphocyte count (LYM) decreased after chemotherapy and RT initially but recovered with maintenance nivolumab ± CIK cell therapy.

Biweekly nivolumab (200 mg, 2.4 mg/kg) was added to the chemotherapy and radiotherapy starting in October 2019. After radiotherapy, S-1 was continued with add-on cytokine-induced killer (CIK) cell therapy, but de-escalated oxaliplatin and nab-paclitaxel were terminated in December 2019 due to severe numbness. Maintenance nivolumab therapy was performed with CIK cell therapy but without S-1 starting in February 2020. Finally, CIK cell therapy was also terminated in December 2020 due to continuing CR. No adverse events were observed with nivolumab therapy and gradual prolongation of the dosing interval (2 → 4 → 6 weeks) due to financial concerns. At present, the patient is still receiving maintenance nivolumab alone every 6 weeks and remains disease-free (**Figure 1C**). The treatment course is summarized in **Figure 2**. Genetic analysis of the biopsy from liver metastasis failed due to an inadequate amount of tissue (immunohistochemistry in the **Supplemental Figures**). However, genetic analysis from peripheral blood mononuclear cells revealed heterozygous mutations of *ATM* (exon4: C283A to Q95K) and *FANCI* (exon29: C3081G to S1027R).

## Discussion

Maintenance therapy in metastatic PDAC has been reported from randomized trials. In the PACT-12 phase II trial, enrolled patients were treated with 6 months of first-line chemotherapy without progression (6). Even without responders, patients receiving maintenance sunitinib had a significantly higher rate of being progression-free at 6 months (PF-6m) than those only under observation (22.2% vs. 3.6%, *P* < 0.01) and better median progression-free survival (PFS; 3.2 vs. 2.0 months, *P* < 0.01) (6). The improvement of median overall survival (OS) was not significant [10.6 vs. 9.2 months; hazard ratio (HR) = 0.71; *P* = 0.11] and was probably related to more than 80% of the patients in both arms receiving second-line therapy at progression (6). By contrast, the phase III POLO trial enrolled patients with metastatic PDAC and a germline *BRCA1* or *BRCA2* mutation under disease control with ≥16 weeks of first-line platinum-based chemotherapy (7). Patients receiving maintenance olaparib had significantly longer median PFS than those on placebo (7.4 vs. 3.8 months; HR = 0.53; *P* = 0.004) as well as a superior response rate (20% vs. 10%) and PF-6m rate (53% vs. 23%) (7). Therefore, the efficacy of maintenance targeted therapy may still rely on a biological match between the drug and the tumor target. Olaparib-based maintenance therapy was not considered in our patient after the withdrawal from the platinum-containing SOLAR regimen because the genetic alterations of the tumor were not initially confirmed. The analyses of germline mutations in the blood sample were requested by the patient and performed during the nivolumab maintenance therapy. Although no germline mutation of *BRCA1* or *BRCA2* was identified, the tumor was highly suspected to have a BRCAness phenotype due to the dramatic response to the platinum-based chemotherapy and alterations in *ATM* and *FANCI*. Considering the supportive data in pancreatic cancer cell lines, targeting pathways involving homologous recombination may be useful as maintenance therapy in patients with *ATM* mutation (8).

For chemosensitive cancers, the concept of maintenance chemotherapy as a de-escalated extension of standard therapy to reduce toxicities is mainly considered in patients with tumors that are adequately stably controlled. By contrast, in advanced PDAC, maintenance chemotherapy is usually overlooked both in real-world practice and clinical trials, probably due to the poor efficacy of the chemotherapy, which results in short OS in the majority of patients. Notably, the prognosis of de-escalated maintenance chemotherapy is still dismal, with limited median PFS after reintroduction of the original regimen at progression (9). The neurotoxicity of oxaliplatin was found to be even higher after FOLFIRINOX was resumed because of a larger cumulative dose comparing to the arm of continuing FOLFIRINOX (9). Cumulative toxicity, such as neurotoxicity, may require management because in more than 40% of patients with metastatic PDAC, progression had not occurred after 6 months of first-line chemotherapy (1, 2). The results of the randomized phase II PANOPTIMOX-PRODIGE 35 trial indicated that use of a maintenance LV5FU2 regimen after disease control with eight cycles of FOLFIRINOX resulted in a comparable median PFS and OS to the complete 12 cycles of FOLFIRINOX (9). However, the highest neurotoxicity occurred later, and the median time to deterioration of quality of life in the maintenance arm was longer (9).

In the present case, after achieving an extreme response to the chemotherapy, gradual dose de-escalation of the chemotherapy combined with local treatment targeting the liver metastasis and pancreatic tumor was adopted to achieve minimal residual disease. Subsequently, gradually de-escalated maintenance immunotherapy was implemented with initially concomitant CIK cell therapy plus nivolumab followed by nivolumab alone. Indeed, a meaningful benefit has rarely been observed when ICIs have been used for blockade of the programmed cell death protein 1 (PD-1)/programmed death ligand 1 pathway in metastatic PDAC without high TMB or dMMR, even in the setting of clinical trials enrolling patients with good PS (3–5). Although information about TMB was not available, the key reason for the durable benefits of maintenance nivolumab with or without CIK cell therapy in our case may have been the balance between the extremely small tumor load and the potentially weak antitumor activity of immunotherapy for PDAC. One phase II study randomized patients with metastatic PDAC who responded or had stable disease (SD) when administered FOLFIRINOX into two groups: those with maintenance granulocyte–macrophage colony-stimulating factor-allogeneic pancreatic tumor cells (GVAX) plus ipilimumab and those for whom FOLFIRINOX was continued (10). No tumor response was observed in patients with GVAX plus ipilimumab, even the beneficial immune reactions in a patient with SD were elicited (10). Median PFS and OS were significantly worse in the immunotherapy arm (10). The failure of this trial may partially reflect the inability of weak maintenance immunotherapy to overcome insufficient prior debulking of tumors with chemotherapy. RFA in our case may have been beneficial for maintenance nivolumab due to direct reduction of the tumor burden. Remodeling of the immune microenvironment at distant non-RFA tumor sites was demonstrated in an animal model (11). As for CIK cell therapy, it is permitted and under the regulation and supervision of the Ministry of Health and Welfare in Taiwan. Infused CIK cells may provide additional source of antitumor cytotoxicity and cytokines to overcome the immunosuppressive tumor microenvironment (12).

## Conclusion

Studies are increasingly investigating ICIs in advanced PDAC, but meaningful benefits have rarely been observed. In conclusion, our case supports further exploration of maintenance nivolumab in patients achieving optimal tumor reduction through multimodality therapy, given the relative safety of anti–PD-1 therapy compared with long-term chemotherapy.

## Data Availability Statement

The original contributions presented in the study are included in the article/**Supplementary Material**. Further inquiries can be directed to the corresponding author.

## Ethics Statement

Ethical review and approval was not required for the study on human participants in accordance with the local legislation and institutional requirements. Written informed consent for participation was not required for this study in accordance with the national legislation and the institutional requirements.

## Author Contributions

Study design: S-HY, S-HK, and CH; Data collection and analysis: S-HY, J-CL, B-BC, and L-YB; Manuscript preparation: S-HY, J-CL, B-BC, and L-YB. All authors contributed to the article and approved the submitted version.

## Conflict of Interest

Ono Pharmaceutical Co., Ltd. supported nivolumab for an ongoing investigator-initiated clinical trial in pancreatic cancer at National Taiwan University Hospital (principal investigator: S-HY).

The remaining authors declare that the research was conducted in the absence of any commercial or financial relationships that could be construed as a potential conflict of interest.

## Publisher’s Note

All claims expressed in this article are solely those of the authors and do not necessarily represent those of their affiliated organizations, or those of the publisher, the editors and the reviewers. Any product that may be evaluated in this article, or claim that may be made by its manufacturer, is not guaranteed or endorsed by the publisher.

## Supplementary Material

Supplementary Figure The immunohistochemical staining of the residual tissues from liver biopsy demonstrated negative staining of CD3, CD4, CD8, CD20, and PD-L1 in the tumor nest.
