# Supplementary figures and images for: Case Report: Maintenance Nivolumab in Complete Responder After Multimodality Therapy in Metastatic Pancreatic Adenocarcinoma
Source: Front Immunol. 2022 Apr 28;13:870406. doi: 10.3389/fimmu.2022.870406 (PMC9097224; doi:10.3389/fimmu.2022.870406)

## Slide 1
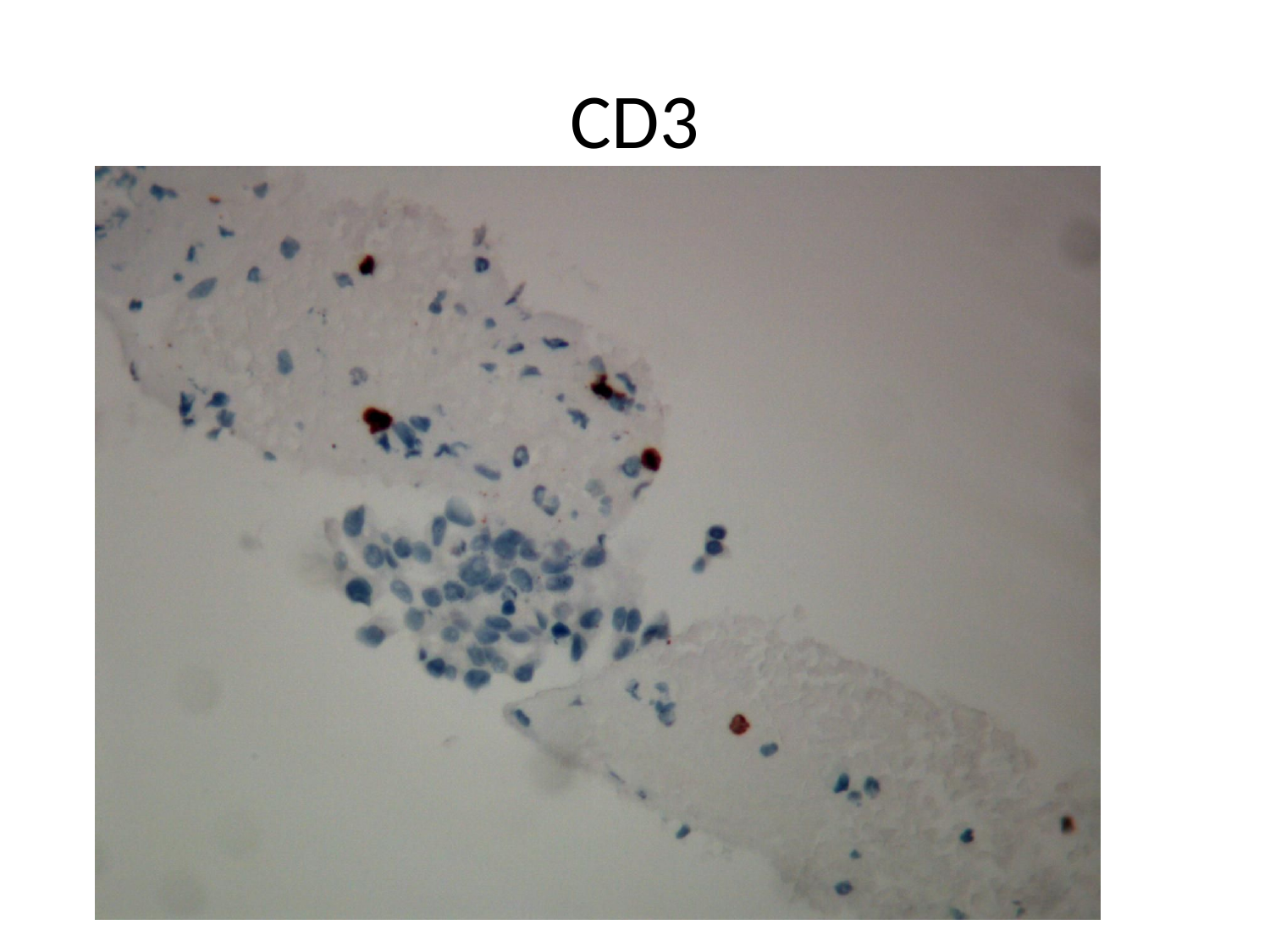

# CD3

## Slide 2
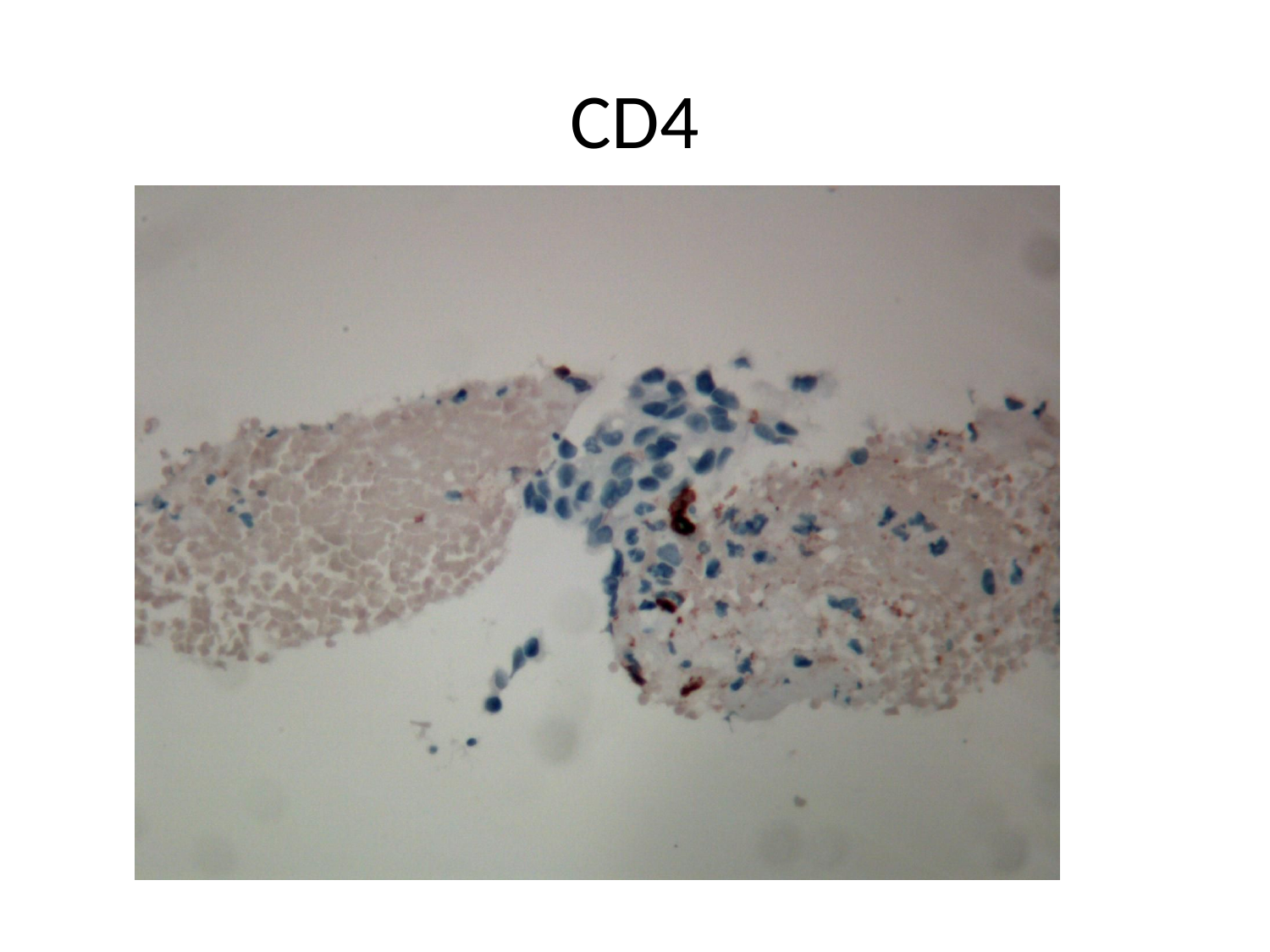

# CD4

## Slide 3
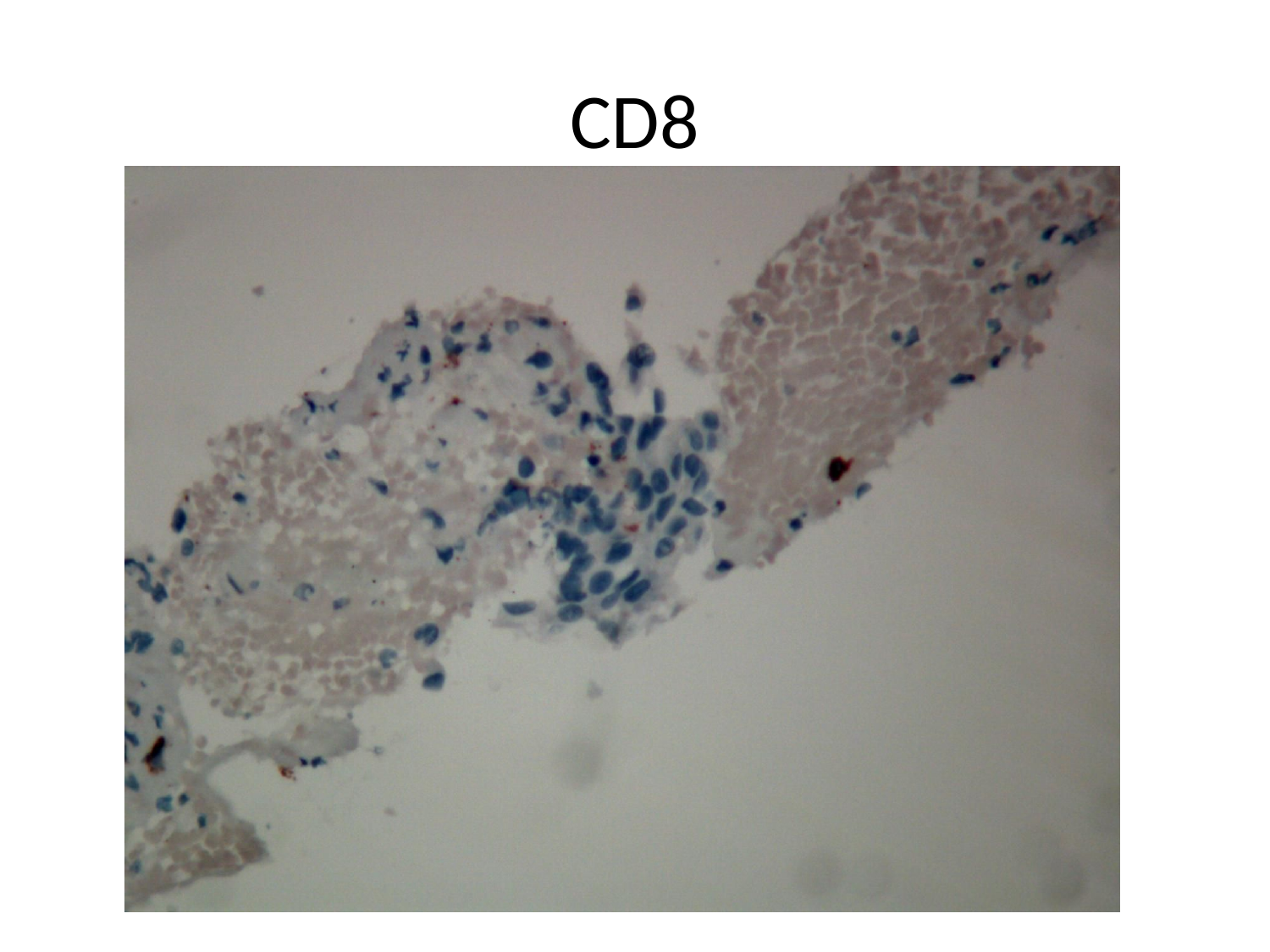

# CD8

## Slide 4
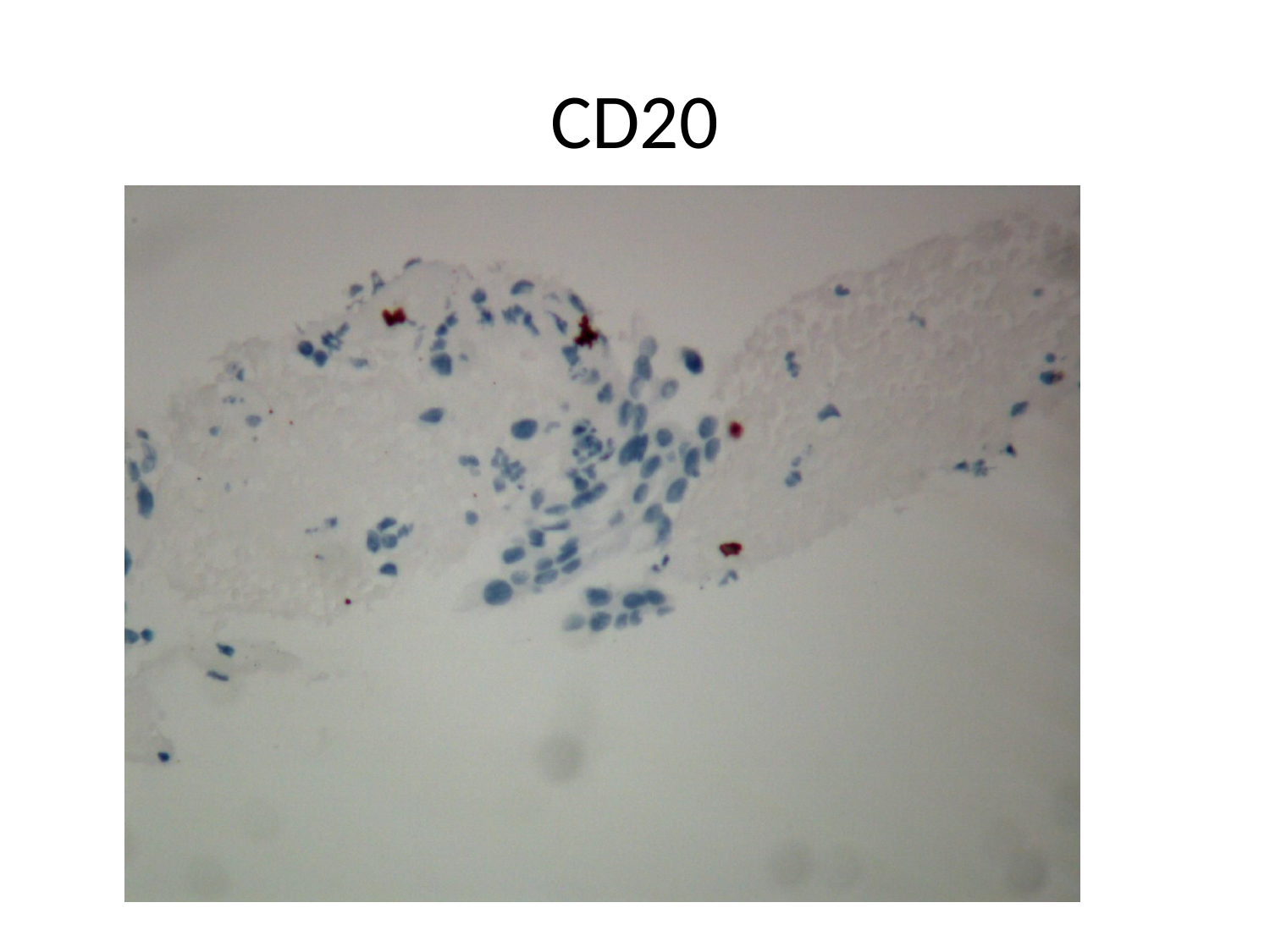

# CD20

## Slide 5
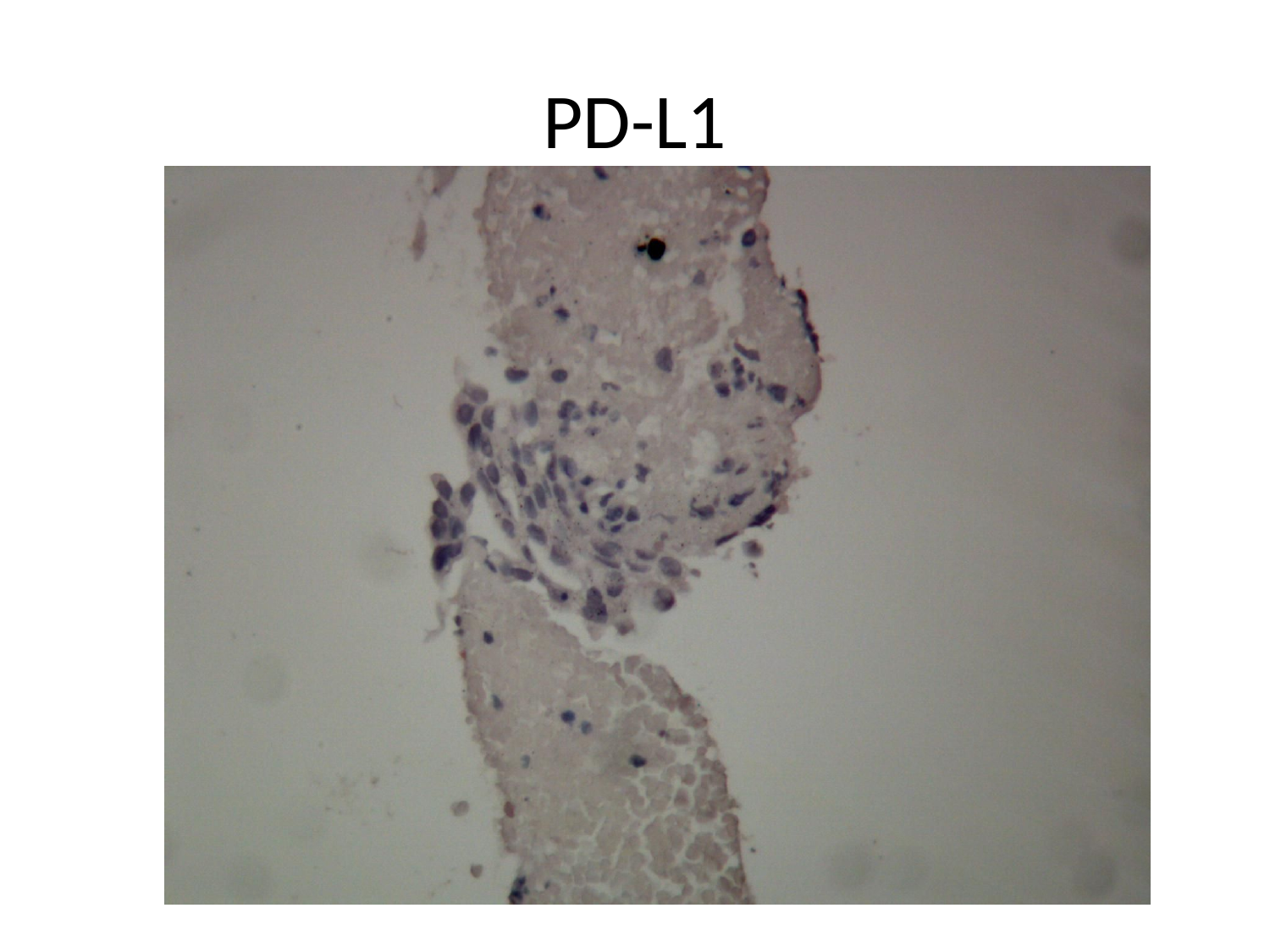

# PD-L1

Supplement: Supplementary Figure — The immunohistochemical staining of the residual tissues from liver biopsy demonstrated negative staining of CD3, CD4, CD8, CD20, and PD-L1 in the tumor nest. [file Presentation_1.pptx]
